# Supplementary material for: A mycobacterial effector promotes ferroptosis-dependent pathogenicity and dissemination
Source: Nat Commun. 2023 Mar 17;14:1430. doi: 10.1038/s41467-023-37148-x (PMC10023711; doi:10.1038/s41467-023-37148-x)
Supplement: Supplementary file 1 — Supplementary Information [file 41467_2023_37148_MOESM1_ESM.pdf]

Supplementary Information

A mycobacterial effector promotes ferroptosis-dependent pathogenicity and dissemination

Lihua Qiang, Yong Zhang, Zehui Lei, Zhe Lu, Shasha Tan, Pupu Ge, Qiyao Chai, Mengyuan Zhao, Xinwen Zhang, Bingxi Li, Yu Pang, Lingqiang Zhang, Cui Hua Liu, Jing Wang

The PDF file includes:

Supplementary Figures 1–8

Supplementary Figure 1

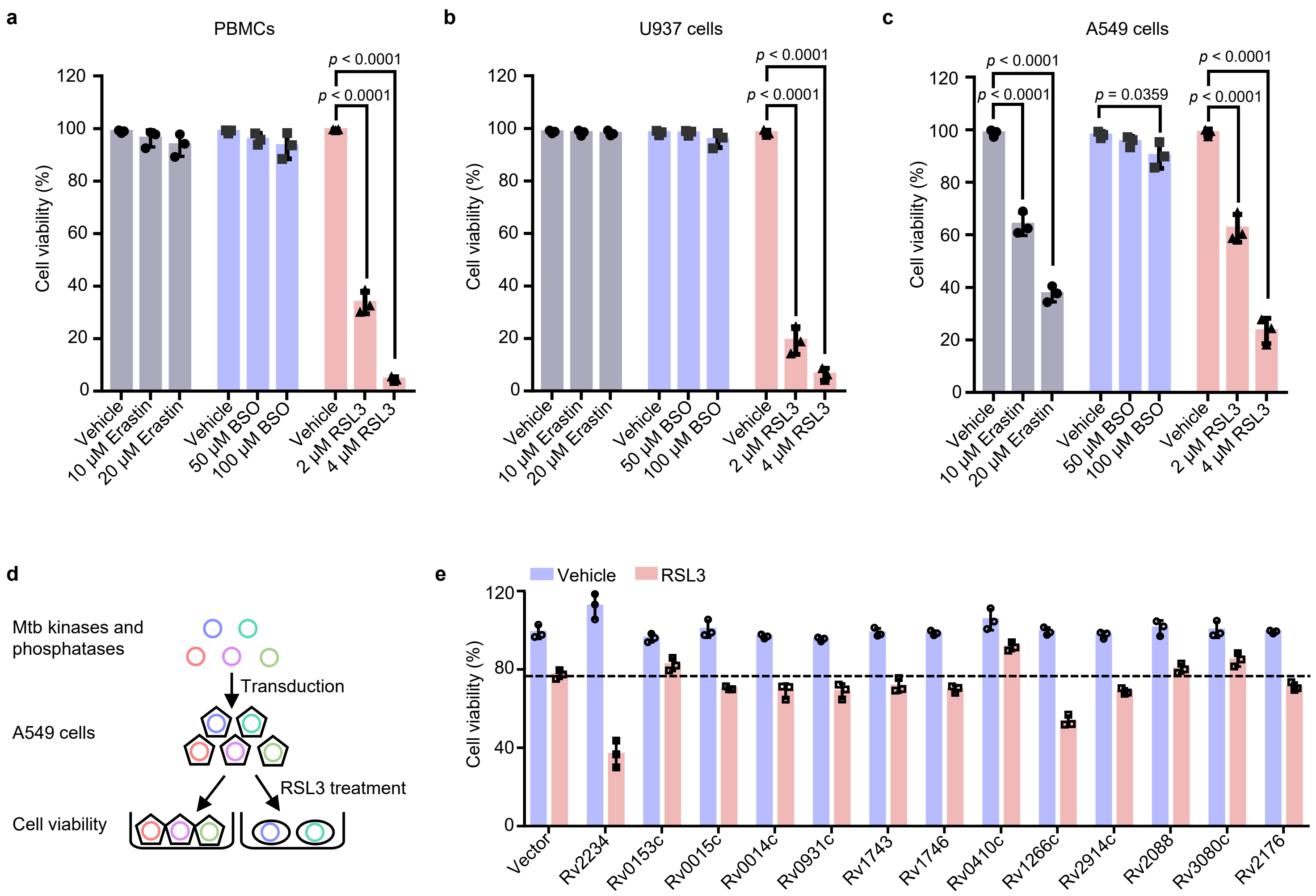

Supplementary Figure 1. Mtb PtpA induces ferroptosis of host cells. a–c Cell viability of PBMCs (**a**), U937 cells (**b**), and A549 cells (**c**) treated with vehicle, Erastin, BSO, or RSL3 for 24 h. **d** Schematic diagram of the procedure to screen Mtb pro-ferroptotic effector in secretory eukaryotic-like phosphatases and kinases. **e** Cell viability (relative to baseline) of A549 cells by Cell Counting Kit-8 (CCK-8) assay. A549 cells were transfected with empty vector or vectors encoding Mtb effector proteins, including secretory eukaryotic-like phosphatases and kinases, for 24 h. Cells were then treated with vehicle or 4 μM RSL3 for 8 h. Error bars are means ± SD of three groups. Statistical significance was determined using two-way ANOVA (Tukey's multiple comparisons test).

Supplementary Figure 2

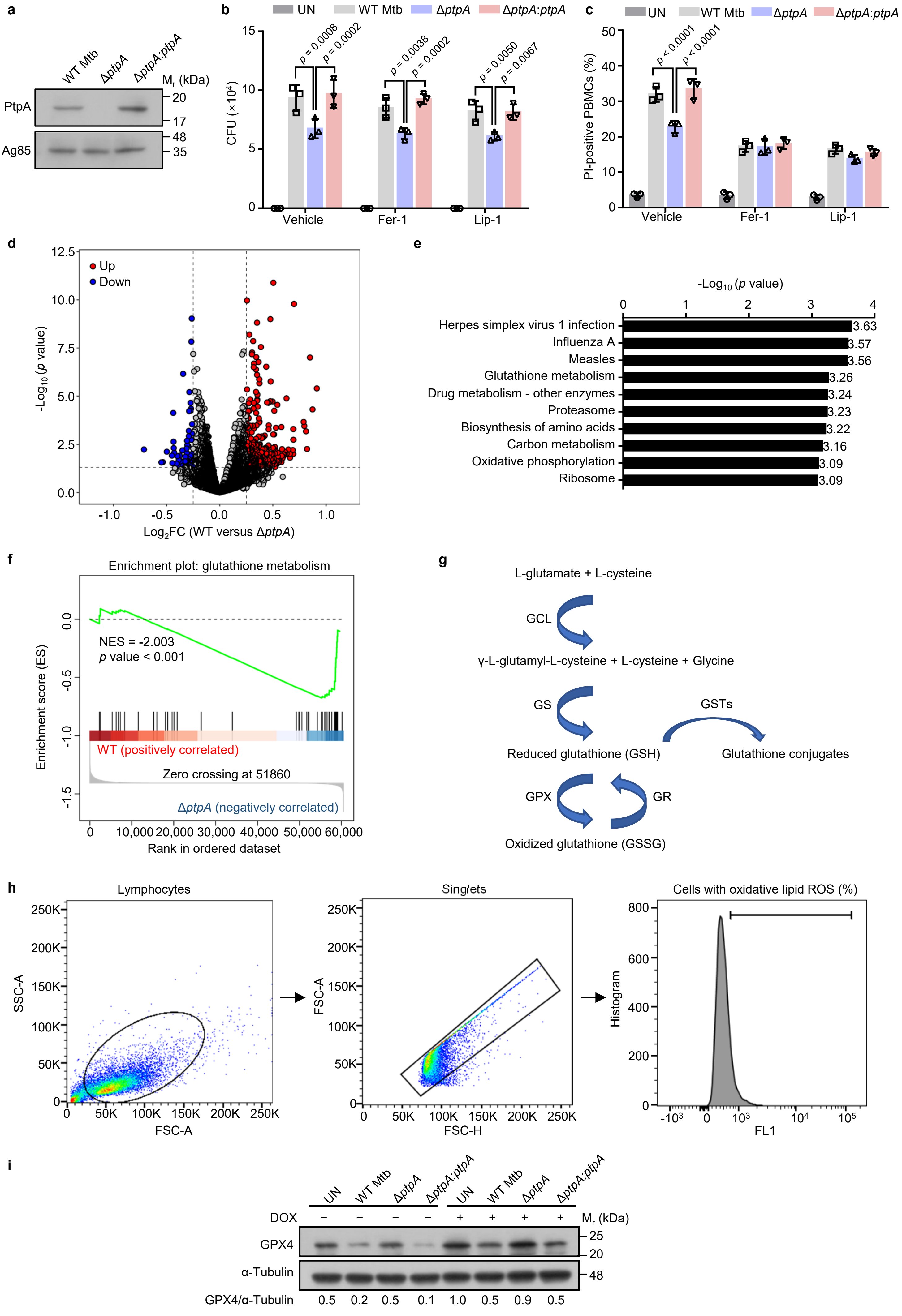

Supplementary Figure 2. Genome-wide analysis links Mtb PtpA to glutathione metabolism-related biological processes. a Immunoblot analysis of PtpA and Ag85 in WT Mtb, Mtb Δ*ptpA*, and Mtb Δ*ptpA*:*ptpA* strains. **b** Survival of Mtb strains in U937 cells. Cells were uninfected (UN) or infected with WT Mtb, Mtb Δ*ptpA*, or Mtb Δ*ptpA*:*ptpA* strain at an MOI of 10 for 24 h with the treatment of vehicle, 10 μM Fer-1, or 10 μM Lip-1. **c** Quantification of PI-positive PBMCs treated as in **b**. **d** Volcano plot for differentially expressed genes in U937 cells infected with WT BCG or *ptpA*-deleted BCG (Δ*ptpA*) strain. The red plot represents up-regulated genes (fold change ≥ 0.25 and p value < 0.05), and the blue plot represents down-regulated genes (fold change ≤ -0.25 and p value < 0.05). **e** The list of the top 10 enriched gene sets. **f** GSEA plot for glutathione metabolism-related gene sets in U937 cells infected with WT BCG or BCG Δ*ptpA* strain. NES, normalized enrichment score. **g** The flow diagram of glutathione metabolism processes in the schema chart. GCL, glutamate cysteine ligase; GS, GSH synthetase; GSTs, glutathione S-transferases; GPX, glutathione peroxidase; GR, glutathione reductase. **h** The gating strategy of flow cytometry. **i** Immunoblot analysis of GPX4 and α-Tubulin in U937 cells. Cells were uninfected or infected with WT Mtb, Mtb Δ*ptpA*, or Mtb Δ*ptpA*:*ptpA* strain at an MOI of 10 for 24 h with or without DOX (1 μM) treatment. Error bars are means ± SD of three groups. Statistical significance was determined using two-way ANOVA (Tukey's multiple comparisons test).

Supplementary Figure 3

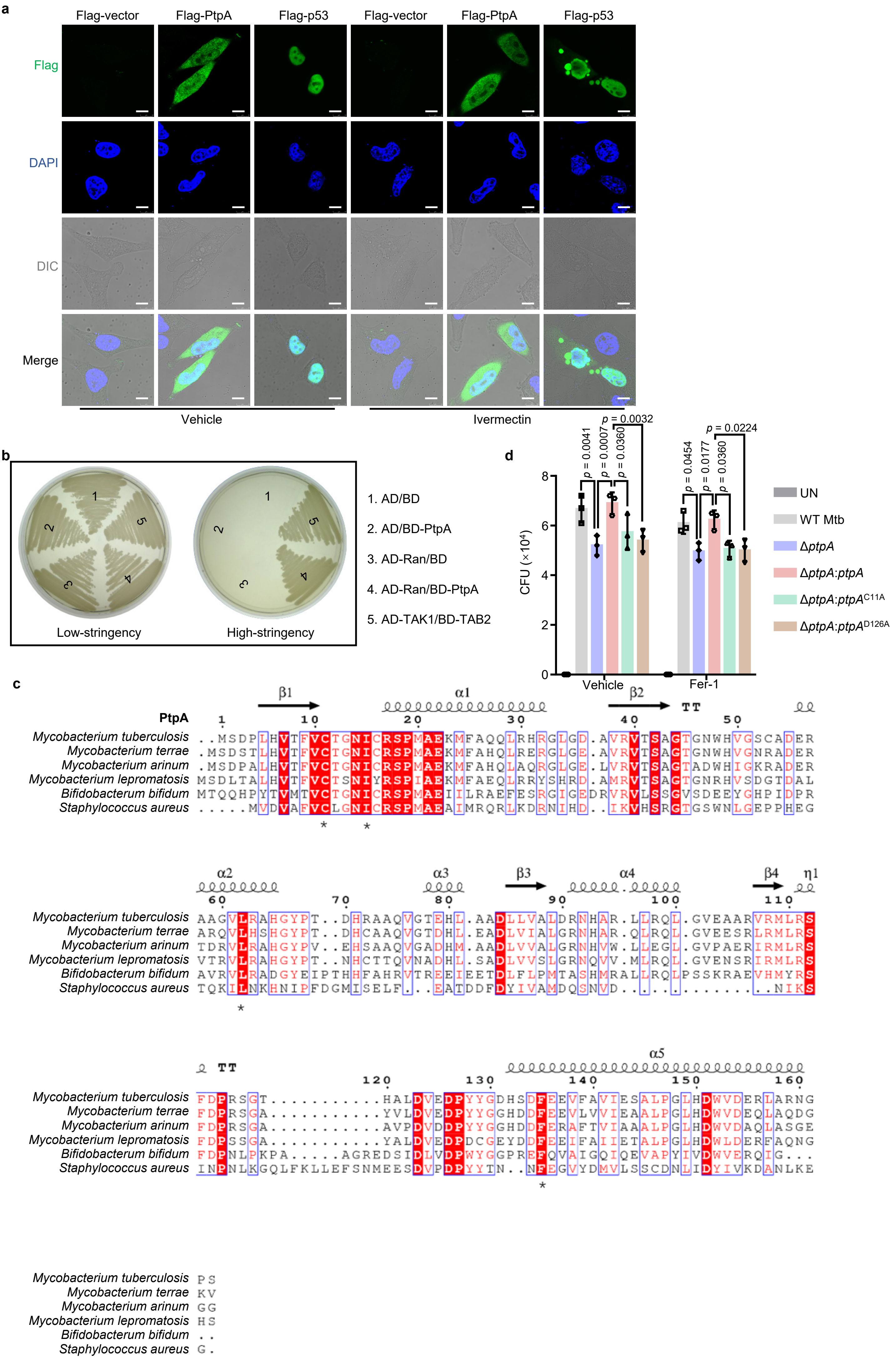

Supplementary Figure 3. PtpA enters host cell nucleus through binding to RanGDP. a Confocal microscopic analysis of the co-localization of PtpA or p53 (a positive control) with host cell nucleus. A549 cells were treated with vehicle or 25 μM ivermectin for 4 h. Flag-tagged proteins (green) were stained with anti-Flag antibody, and nuclei (blue) were stained with DAPI. Scale bars, 10 μm. **b** The interaction assay between PtpA and Ran GTPase using yeast two-hybrid system. The transformants were plated onto low-stringency (left) and high-stringency (right) selection plates, respectively. **c** Multiple sequence alignment of PtpA from Mtb, *Mycobacterium terrae*, *Mycobacterium marinum*, *Mycobacterium lepromatosis*, *Bifidobacterium bifidum*, and *Staphylococcus aureus*, using ESPript 3.0 server (https://espript.ibcp.fr/ESPript/cgi-bin/ESPript.cgi). Conserved residues and conserved hydrophobic residues are marked with red background and asterisk, respectively. **d** Survival of Mtb strains in U937 cells. Cells were uninfected or infected with WT Mtb, Mtb Δ*ptpA*, Mtb Δ*ptpA*:*ptpA*, Mtb Δ*ptpA*:*ptpA*^{C11A}, or Mtb Δ*ptpA*:*ptpA*^{D126A} strain at an MOI of 10 for 24 h with the treatment of vehicle or 10 μM Fer-1. Error bars are means ± SD of three groups. Statistical significance was determined using two-way ANOVA (Tukey’s multiple comparisons test).

Supplementary Figure 4

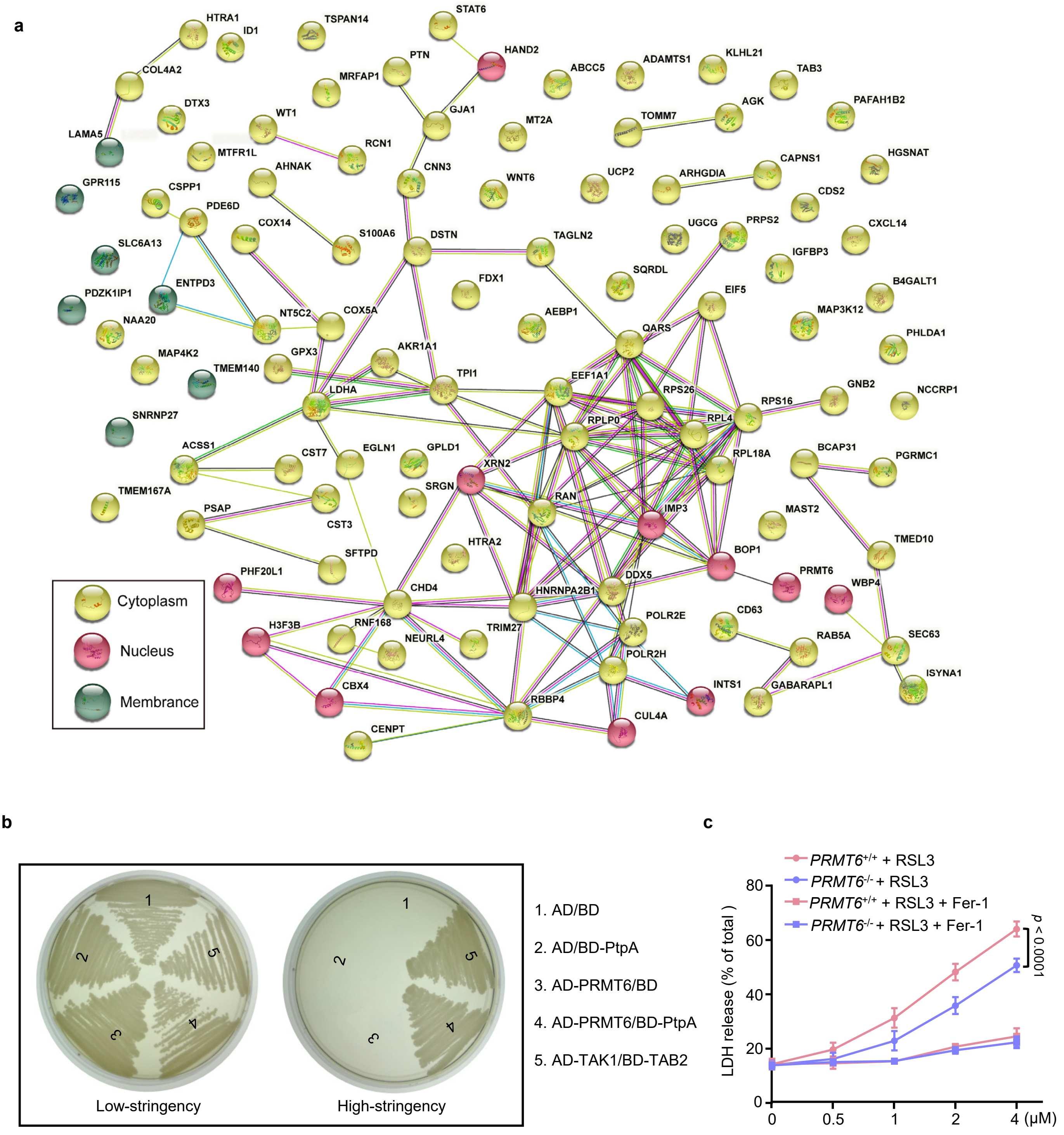

Supplementary Figure 4. Mtb PtpA interacts with PRMT6. a Protein-protein interaction network of PtpA-interacting host proteins screened by yeast two-hybrid system. The interaction overlap and subcellular localization of PtpA-interacting host proteins were predicted using STRING (https://string-db.org). **b** The interaction assay between PtpA and PRMT6 using yeast two-hybrid system. The transformants were plated onto low-stringency (left) and high-stringency (right) selection plates, respectively. **c** LDH release of *PRMT6*$^{+/+}$ and *PRMT6*$^{-/-}$ U937 cells untreated or treated with 0.5, 1, 2, or 4 μM RSL3 for 8 h, in the presence or absence of 10 μM Fer-1. Error bars are means ± SD of three groups. Statistical significance was determined using two-way ANOVA (Tukey's multiple comparisons test).

Supplementary Figure 5

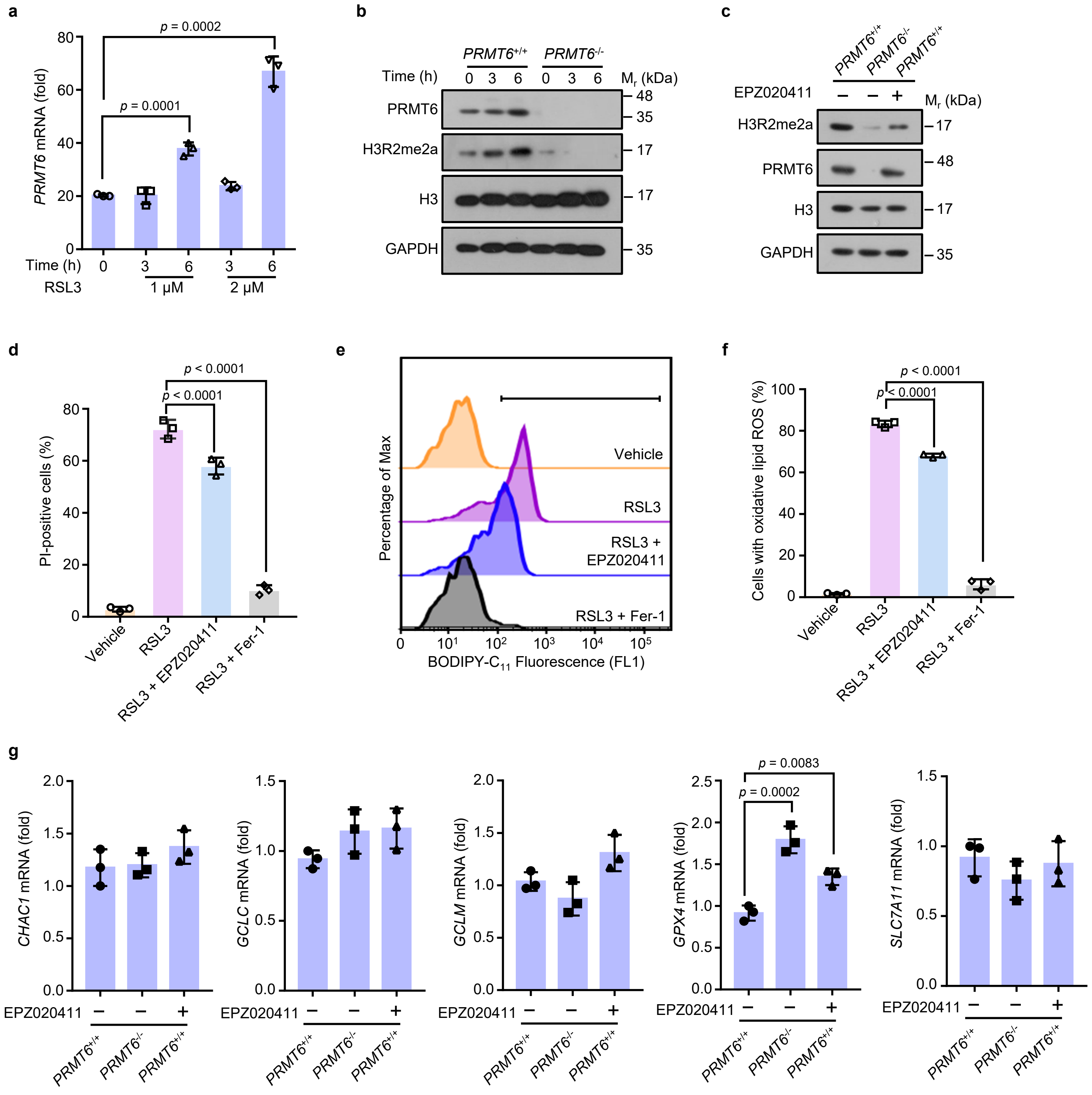

Supplementary Figure 5. PRMT6 is involved in ferroptosis regulation depending on its methyltransferase activity. a RT-qPCR analysis of *PRMT6* mRNA in U937 cells. Cells were untreated or treated with 1 or 2 μM RSL3 for 3 h or 6 h. **b** Immunoblot analysis of PRMT6, H3R2me2a, H3, and GAPDH in *PRMT6*$^{+/+}$ and *PRMT6*$^{-/-}$ U937 cells. Cells were treated with 2 μM RSL3 for 0 h, 3 h, or 6 h. **c** Immunoblot analysis of H3R2me2a, PRMT6, H3, and GAPDH in *PRMT6*$^{+/+}$ U937 cells, *PRMT6*$^{-/-}$ U937 cells, and *PRMT6*$^{+/+}$ U937 cells treated with 10 μM EPZ020411 for 24 h. **d** Quantification of PI-positive U937 cells. Cells were divided into four groups: vehicle, RSL3, RSL3 + EPZ0204111, and RSL3 + Fer-1. For vehicle, RSL3, and RSL3 + Fer-1 groups, U937 cells were treated with vehicle, 2 μM RSL3, or 2 μM RSL3 plus 10 μM Fer-1 for 8 h. For RSL3 + EPZ0204111 group, U937 cells were treated with 10 μM EPZ020411 for 24 h and with RSL3 for additional 8 h. **e** Flow cytometry analysis of U937 cells with oxidative lipid ROS. Cells were treated as in **d**, and marked with BODIPY C_{11} lipid probe. **f** Quantification of U937 cells with oxidative lipid ROS. Cells were treated as in **e**. **g** RT-qPCR analysis of *CHAC1* mRNA, *GCLC* mRNA, *GCLM* mRNA, *GPX4* mRNA, and *SLC7A11* mRNA in *PRMT6*$^{+/+}$ U937, *PRMT6*$^{-/-}$ U937, and *PRMT6*$^{+/+}$ U937 cells treated with 10 μM EPZ020411 for 24 h. Error bars are means ± SD of three groups. Statistical significance was determined using one-way ANOVA (Dunnett's multiple comparisons test).

Supplementary Figure 6

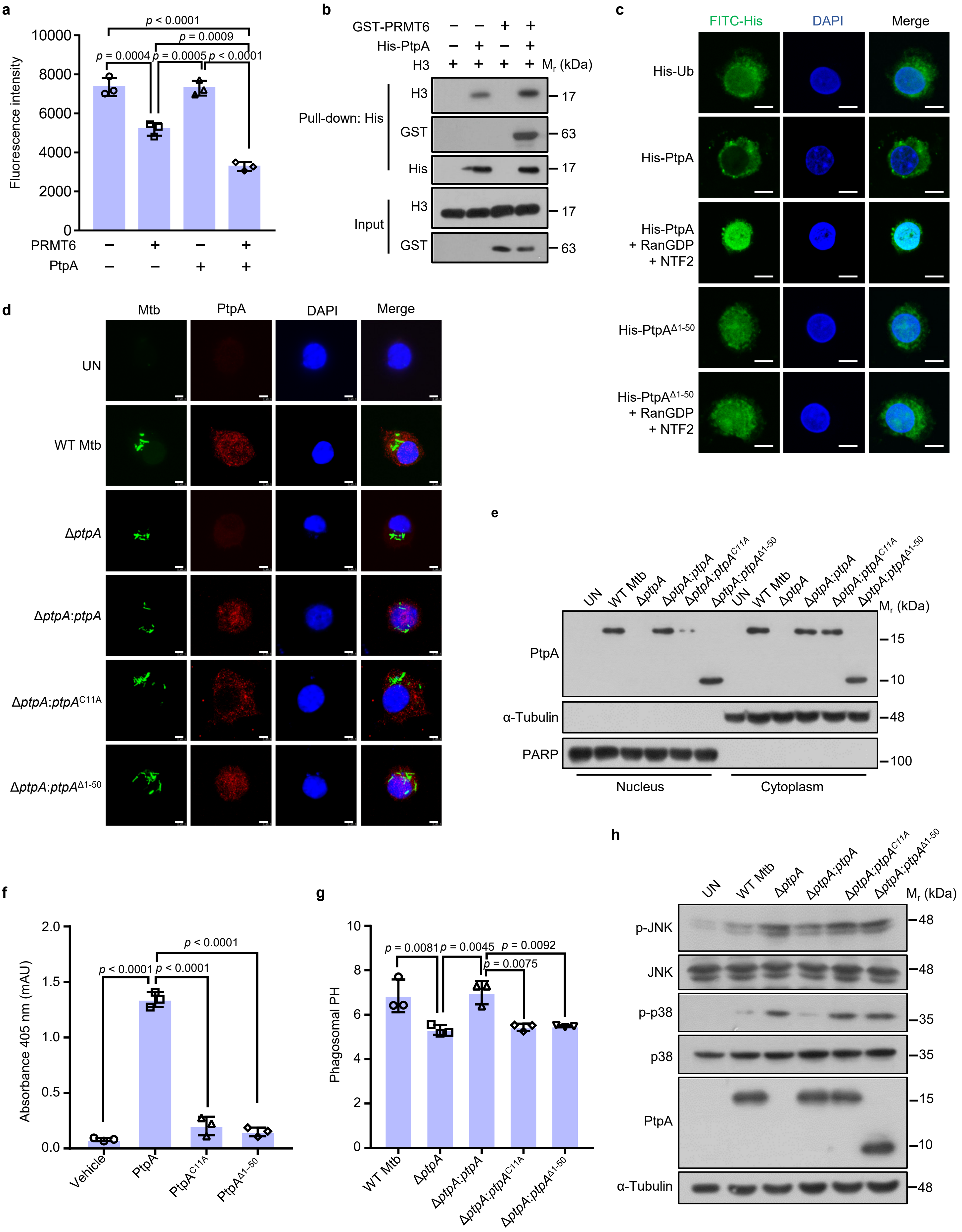

Supplementary Figure 6. Mtb PtpA enhances the methyltransferase activity of PRMT6. **a** Fluorescence intensity of Bz-Arg-AMC in the presence of PRMT6, PtpA, or PRMT6 plus PtpA. **b** Pull-down assay of H3 and GST-PRMT6 by His-PtpA. **c** Confocal microscopic analysis of protein nuclear localization using digitonin-treated semipermeable U937 cells. Cells were incubated with His-Ub, His-PtpA, or His-PtpA$^{\Delta 1\text{-}50}$ with or without GST-RanGDP and GST-NTF2. His-tagged proteins (green) were stained with anti-His antibody, and nuclei (blue) were stained with DAPI. Scale bar, 5 µm. **d** Confocal microscopic analysis for nuclear localization of PtpA, PtpA^{C11A}, or ptpA$^{\Delta 1\text{–}50}$ in U937 cells. Cells were uninfected or infected with WT Mtb, Mtb Δ*ptpA*, Mtb Δ*ptpA*:*ptpA*, Mtb Δ*ptpA*:*ptpA*^{C11A}, or Mtb Δ*ptpA*:*ptpA*$^{\Delta 1\text{–}50}$ strain at an MOI of 10 for 8 h. Mtb strains were stained with Alexa Fluor 488 succinimidyl ester, PtpA or its mutants (red) were stained with anti-PtpA antibody, and nuclei (blue) were stained with DAPI. Scale bar, 5 µm. **e** Immunoblot analysis of the cytosolic fraction and the pellets containing the nuclei in U937 cells. Cells were uninfected or infected with WT Mtb, Mtb Δ*ptpA*, Mtb Δ*ptpA*:*ptpA*, Mtb Δ*ptpA*:*ptpA*^{C11A}, or Mtb Δ*ptpA*:*ptpA*$^{\Delta 1\text{–}50}$ strain at an MOI of 20 for 8 h. **f** Phosphatase activity of PtpA, PtpA^{C11A}, and PtpA$^{\Delta 1\text{–}50}$ analyzed by para-nitrophenyl phosphate (pNPP) phosphatase assay. **g** Phagosomal pH of the infected U937 cells was measured with fluorescence activated cell sorting (FACS). WT Mtb, Mtb Δ*ptpA*, Mtb Δ*ptpA*:*ptpA*, Mtb Δ*ptpA*:*ptpA*^{C11A}, or Mtb Δ*ptpA*:*ptpA*$^{\Delta 1\text{–}50}$ strain were labeled with pH-sensitive fluorescent dye (pHrodo) and used to infect U937 cells at an MOI of 10 for 4 h. **h** Immunoblot analysis of p-JNK, JNK, p-p38, p38, PtpA, and α-Tubulin in U937 cells infected with the indicated Mtb strains for 4 h as in **e**. Error bars are means ± SD of three groups. Statistical significance was determined using one-way ANOVA (Dunnett's multiple comparisons test).

Supplementary Figure 7

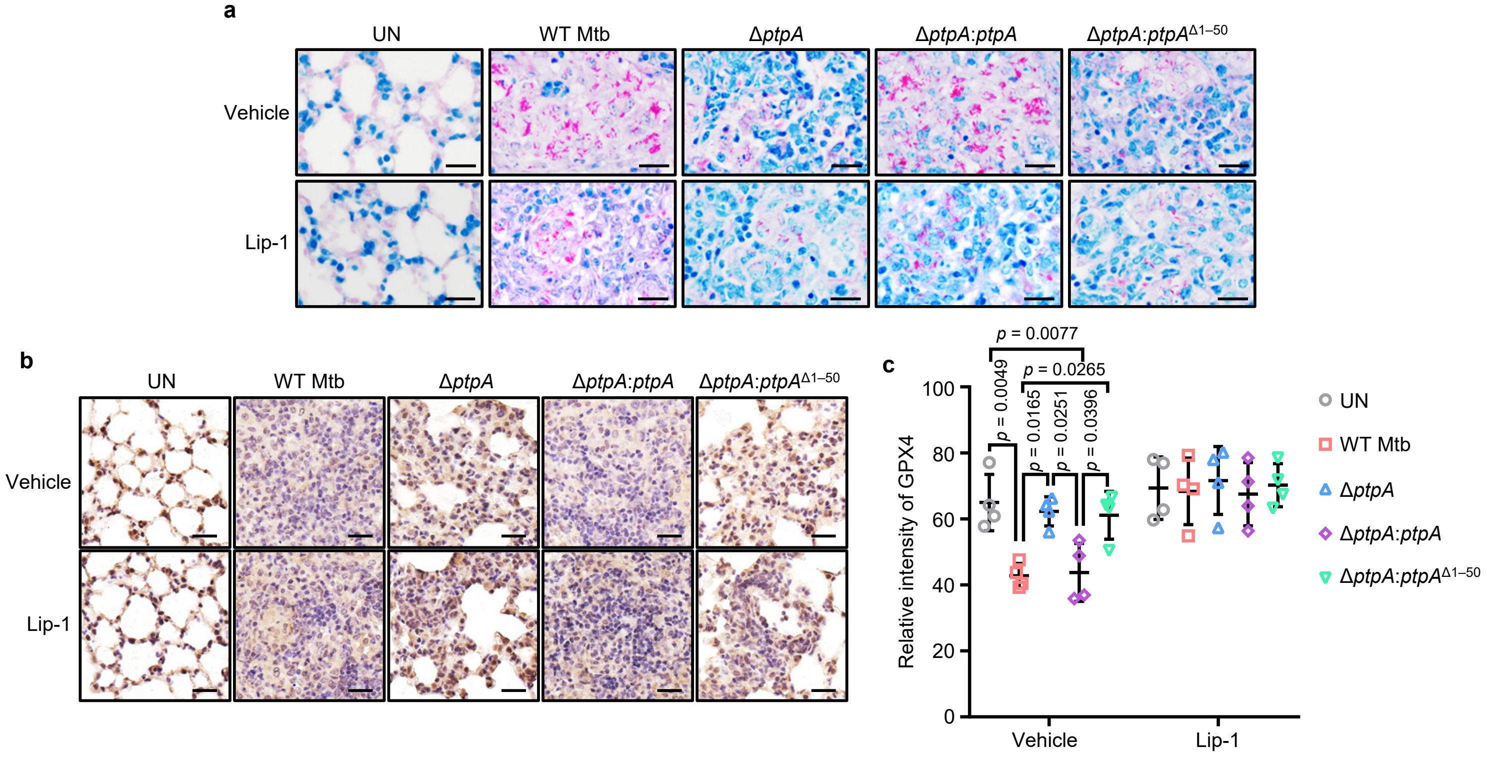

Supplementary Figure 7. Lip-1 treatment reduces bacteria burden and promotes GPX4 expression in mice. **a** Acid-fast staining of lung sections from mice infected with WT Mtb, Mtb Δ*ptpA*, Mtb Δ*ptpA*:*ptpA*, or Mtb Δ*ptpA*:*ptpA*$^{\Delta 1-50}$ strain for 5 weeks. Uninfected mice were served as the control. Daily intraperitoneal injections of vehicle or Lip-1 (3 mg/kg) were administered to uninfected or infected mice from day 15 after Mtb infection (n = 4). Scale bars, 20 μm. **b** Immunohistochemical staining of GPX4 in lung sections from mice as in **a**. Scale bars, 20 μm. **c** Quantitative analysis of GPX4 expression in lungs from mice as in **a.** Error bars are means ± SD of four mice per group and each represents data from two separate experiments. Statistical significance was determined using two-way ANOVA (Tukey's multiple comparisons test).

Supplementary Figure 8

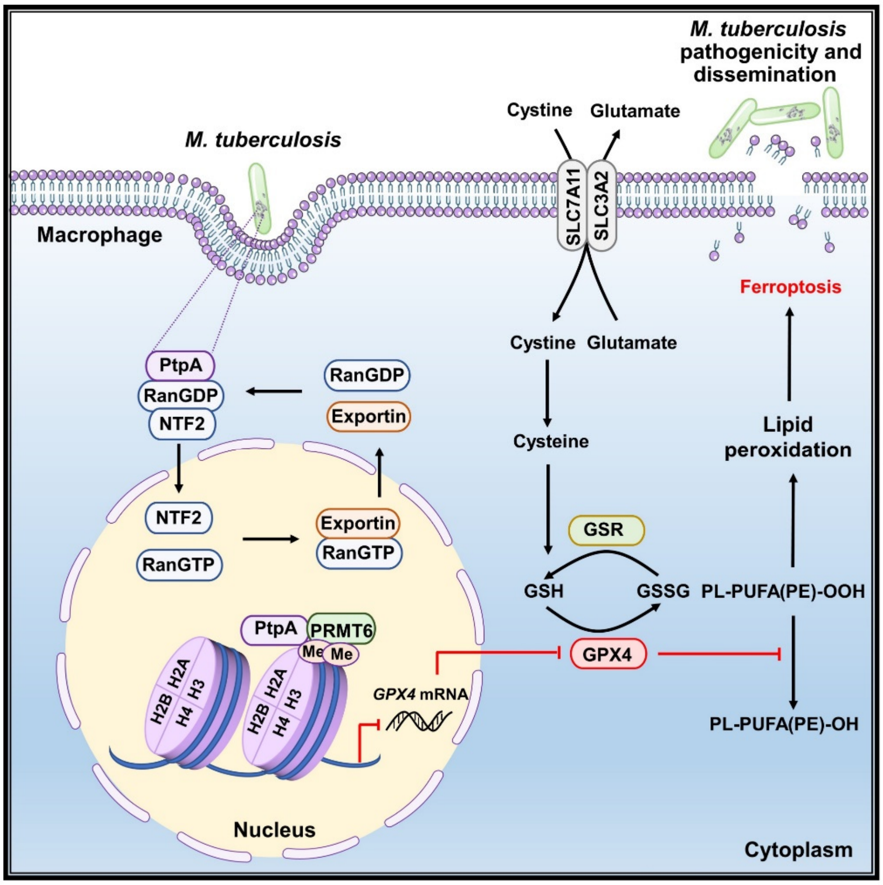

Supplementary Figure 8. Schematic model shows that nuclear PtpA enhances PRMT6-mediated H3R2me2a to promote ferroptosis-dependent pathogen pathogenicity and dissemination. Briefly, Mtb effector PtpA enters host cell nucleus to enhance PRMT6-mediated asymmetric demethylation of H3R2, leading to the inhibition of GPX4 expression, followed by promoting ferroptosis-dependent pathogen pathogenicity and dissemination.
