# Supplementary material for: A mycobacterial effector promotes ferroptosis-dependent pathogenicity and dissemination
Source: Nat Commun. 2023 Mar 17;14:1430. doi: 10.1038/s41467-023-37148-x (PMC10023711; doi:10.1038/s41467-023-37148-x)
Supplement: Supplementary file 3 — Description of Additional Supplementary Files [file 41467_2023_37148_MOESM3_ESM.pdf]

Description of Additional Supplementary Files

Supplementary Data 1. Plasmids and oligonucleotides. Lists of the names, descriptions, and sources of plasmids and oligonucleotides used in this study.

Supplementary Movie 1. Cell viability of WT U937 cells treated with RSL3. Cells were treated with 2 μM RSL3 and were then incubated with 5 μg/mL propidium iodide (PI). Images were acquired in 5 min intervals over a time frame of 6 h. Dead cells (red) were stained with PI. Scale bars, 25 μm.

Supplementary Movie 2. Cell viability of U937 cells stably expressing PtpA treated with RSL3. Cells were treated with 2 μM RSL3 and were then incubated with 5 μg/mL PI. Images were acquired in 5 min intervals over a time frame of 6 h. Dead cells (red) were stained with PI. Scale bars, 25 μm.
