# Supplementary material for: A mycobacterial effector promotes ferroptosis-dependent pathogenicity and dissemination
Source: Nat Commun. 2023 Mar 17;14:1430. doi: 10.1038/s41467-023-37148-x (PMC10023711; doi:10.1038/s41467-023-37148-x)
Supplement: Supplementary file 7 — Reporting Summary [file 41467_2023_37148_MOESM7_ESM.pdf]

nature portfolio

Corresponding author(s): Jing Wang

Last updated by author(s): Feb 7, 2023

Reporting Summary

Nature Portfolio wishes to improve the reproducibility of the work that we publish. This form provides structure for consistency and transparency in reporting. For further information on Nature Portfolio policies, see our Editorial Policies and the Editorial Policy Checklist.

Statistics

For all statistical analyses, confirm that the following items are present in the figure legend, table legend, main text, or Methods section.

n/a	Confirmed	
☐	☒	The exact sample size (*n*) for each experimental group/condition, given as a discrete number and unit of measurement
☐	☒	A statement on whether measurements were taken from distinct samples or whether the same sample was measured repeatedly
☐	☒	The statistical test(s) used AND whether they are one- or two-sided<br>*Only common tests should be described solely by name; describe more complex techniques in the Methods section.*
☒	☐	A description of all covariates tested
☐	☒	A description of any assumptions or corrections, such as tests of normality and adjustment for multiple comparisons
☐	☒	A full description of the statistical parameters including central tendency (e.g. means) or other basic estimates (e.g. regression coefficient) AND variation (e.g. standard deviation) or associated estimates of uncertainty (e.g. confidence intervals)
☐	☒	For null hypothesis testing, the test statistic (e.g. *F*, *t*, *r*) with confidence intervals, effect sizes, degrees of freedom and *P* value noted<br>*Give P values as exact values whenever suitable.*
☒	☐	For Bayesian analysis, information on the choice of priors and Markov chain Monte Carlo settings
☒	☐	For hierarchical and complex designs, identification of the appropriate level for tests and full reporting of outcomes
☒	☐	Estimates of effect sizes (e.g. Cohen's *d*, Pearson's *r*), indicating how they were calculated

Our web collection on statistics for biologists contains articles on many of the points above.

Software and code

Policy information about availability of computer code

Data collection	Quantitative PCR: ABI 7500 system.<br>Immunohistochemistry: Leica CS2.<br>Immunofluorescence: Leica SP8 Biosystems.<br>Flow cytometry: BD LSRFortessa SORP, BD FACSAria III.
Data analysis	Image analysis: ImageJ (v1.8.0) software, SlideViewer (v2.5.0.143918), ImageScope (v12.3.3.7014), Leica Application Suite Las X (v2.0.1.14392).<br>Statistical analysis: GraphPad Prism (v8.0).<br>Flow cytometric analysis: FlowJo (v10).<br>RNA-sequencing analysis: FASTQC (v0.11.3), STAR (v2.4.2a), R (v3.4.4), DEseq2 (v1.18.1).

For manuscripts utilizing custom algorithms or software that are central to the research but not yet described in published literature, software must be made available to editors and reviewers. We strongly encourage code deposition in a community repository (e.g. GitHub). See the Nature Portfolio guidelines for submitting code & software for further information.

Data

Policy information about availability of data

All manuscripts must include a data availability statement. This statement should provide the following information, where applicable:

- Accession codes, unique identifiers, or web links for publicly available datasets
- A description of any restrictions on data availability
- For clinical datasets or third party data, please ensure that the statement adheres to our policy

The RNA-sequencing data have been deposited in NCBI Gene Expression Omnibus database under accession number GSE199069 (https://www.ncbi.nlm.nih.gov/geo/query/acc.cgi?acc=GSE199069), and these data have been publicly released. The amino acid sequence was analyzed in the SMART database (https://smart.embl.de/smart/show_motifs.pl). All the data supporting the findings of this study are available within the article and its Supplementary Information. Source data are provided with this paper.

Human research participants

Policy information about studies involving human research participants and Sex and Gender in Research.

Reporting on sex and gender	A total of two healthy donors (including 1 female and 1 male) were recruited for collecting peripheral blood mononuclear cells (PBMCs) to analyze the susceptibility of human primary cells to ferroptosis (Supplementary Fig. 1a), which was not affected by sex or gender. Thus, the experiment used PBMCs in this study didn't perform sex- or gender- based analyses.
Population characteristics	The individuals without known history of exposure to Mycobacterium tuberculosis were included as healthy donors based on negative result of T- SPOT.TB test (a type of interferon gamma release assay used for tuberculosis diagnosis). The female donor is 30 years old and the male donor is 28 years old. All participants have no significant other medical history such as human immunodeficiency virus (HIV) infection, cancers and diabetes.
Recruitment	All participants were recruited randomly for whole blood donation at Beijing Chest Hospital, Capital Medical University. Donors were required to sign an informed consent document before participating.
Ethics oversight	Ethical permission for this study was obtained from the ethics committee of Beijing Chest Hospital, Capital Medical University, Beijing, China.

Note that full information on the approval of the study protocol must also be provided in the manuscript.

Field-specific reporting

Please select the one below that is the best fit for your research. If you are not sure, read the appropriate sections before making your selection.

☒ Life sciences ☐ Behavioural & social sciences ☐ Ecological, evolutionary & environmental sciences

For a reference copy of the document with all sections, see nature.com/documents/nr-reporting-summary-flat.pdf

Life sciences study design

All studies must disclose on these points even when the disclosure is negative.

Sample size	All sample sizes are indicated in the figure legends. No sample size calculation was performed. To determine sample sizes in vitro, the number of samples that were available and the number of samples required to establish statistical significance were taken into consideration. For in vivo, the sample size was determined based on our previous work (e.g. Wang et al., Nat Commun, 2017; Wang et al., Nat Immunol, 2015).
Data exclusions	No data were excluded.
Replication	The number of replicates for all experiments are indicated in the figure legends. All experiments could be successfully replicated and showed comparable results.
Randomization	For in vivo study, each group of four mice with similar ages were randomly allocated into different groups. For in vitro study, no formal randomization method was used, because the experimental treatments were distributed equally among all groups for each experiment.
Blinding	For in vitro study, investigators were not blinded to the sample identities during data collection since the readouts were quantitative and not prone to subjective judgment of investigators. For in vivo study, mice experiments and statistical analysis were performed by independent researchers in a blinded manner.

Reporting for specific materials, systems and methods

We require information from authors about some types of materials, experimental systems and methods used in many studies. Here, indicate whether each material, system or method listed is relevant to your study. If you are not sure if a list item applies to your research, read the appropriate section before selecting a response.

Materials & experimental systems

n/a	Involved in the study	
☐	☒	Antibodies
☐	☒	Eukaryotic cell lines
☒	☐	Palaeontology and archaeology
☐	☒	Animals and other organisms
☒	☐	Clinical data
☒	☐	Dual use research of concern

Methods

n/a	Involved in the study	
☒	☐	ChIP-seq
☐	☒	Flow cytometry
☒	☐	MRI-based neuroimaging

Antibodies

Antibodies used

All of the antibodies were used according to the manufacturer's instructions and based on previous experience in the laboratory. The following commercial antibodies have been used:
Anti-Flag antibody, 1:200 for immunofluorescence, Sigma-Aldrich, Cat# F1804;
Anti-Ag85 antibody, 1:1000 for immunoblotting, Abcam, Cat# ab36731;
Anti-Mtb antibody, 1:200 for immunofluorescence, Abcam, Cat# ab905;
Anti-DDDDK-tag-pAb-HRP-DirecT, 1:2000 for immunoblotting, Medical & biological laboratories, Cat# PM020-7;
Anti-GFP-tag-pAb-HRP-DirecT, 1:1000 for immunoblotting, Medical & biological laboratories, Cat# 598-7;
Anti-H3R2me2a antibody, 1:1000 for immunoblotting, Abcam, Cat# ab194706;
Anti-H3R2me2a antibody, 1:200 for immunofluorescence, ABclonal, Cat# A3155;
Anti-PRMT6 antibody, 1:1000 for immunoblotting, Cell Signaling Technologies, Cat# 14641S;
Anti-H3 antibody, 1:1000 for immunoblotting, Abcam, Cat# ab1791;
Anti-α-Tubulin antibody, 1:2000 for immunoblotting, Sigma-Aldrich, Cat# T6199;
Anti-GPX4 antibody, 1:1000 for immunoblotting and 1:200 immunohistochemistry, ABclonal, Cat# A11243;
Anti-GST antibody, 1:1000 for immunoblotting, ABclonal, Cat# AE006;
Anti-PARP antibody, 1:1000 for immunoblotting, Cell Signaling Technologies, Cat# 9542;
Anti-His antibody, 1:1000 for immunoblotting, ABclonal, Cat# AE003;
Anti-4 Hydroxynonenal antibody, 1:200 for immunohistochemistry, Abcam, Cat# ab46545;
Anti-GAPDH antibody, 1:5000 for immunoblotting, Santa Cruz, Cat# sc-25778;
Anti-JNK antibody, 1:1000 for immunoblotting, Cell Signaling Technologies, Cat# 9252;
Anti-p-JNK antibody, 1:1000 for immunoblotting, Cell Signaling Technologies, Cat# 9255;
Anti-p38 antibody, 1:1000 for immunoblotting, Cell Signaling Technologies, Cat# 9212;
Anti-p-p38 antibody, 1:1000 for immunoblotting, Cell Signaling Technologies, Cat# 9211;
Goat-anti-Mouse HRP, 1:10000 for immunoblotting, ZSGB-BIO, Cat# ZB-2305;
Goat-anti-Rabbit HRP, 1:10000 for immunoblotting, ZSGB-BIO, Cat# ZB-2306;
Goat-anti-Mouse Alexa Fluor 594, 1:200 for immunofluorescence, ZSGB-BIO, Cat# ZF-0513;
Goat-anti-Mouse FITC, 1:200 for immunofluorescence, ZSGB-BIO, Cat# ZF-0312;
Goat anti-Mouse IgG (H+L) Secondary Antibody, DyLight 405, 1:200 for immunofluorescence, Invitrogen, Cat# 35501BID.
Anti-PtpA antibody was produced and purified by GenScript Biotechnology with the recombinant GST-tagged PtpA protein as the immunogen, 1:1000 for immunoblotting and1:200 for immunofluorescence.

Validation

Antibodies used in this study are commercially available and are validated by the manufacturers. The related information are available on their manufacturer's website:
Anti-Flag antibody, Cat# F1804, https://www.sigmaaldrich.cn/CN/zh/product/sigma/f1804;
Anti-Ag85 antibody, Cat# ab36731, https://www.abcam.cn/mycobacterium-tuberculosis-ag85-antibody-hyt27-ab36731.html;
Anti-Mtb antibody, Cat# ab905, https://www.abcam.cn/mycobacterium-tuberculosis-antibody-ab905.html;
Anti-DDDDK-tag-pAb-HRP-DirecT, Cat# PM020-7, https://www.mblbio.com/bio/g/dtl/A/?pcd=PM020-7;
Anti-GFP-tag-pAb-HRP-DirecT, Cat# 598-7, https://www.mblbio.com/bio/g/dtl/A/?pcd=598-7;
Anti-H3R2me2a antibody, Cat# ab194706, https://www.abcam.cn/histone-h3-asymmetric-di-methyl-r2-antibody-ab194706.html;
Anti-H3R2me2a antibody, Cat# A3155, https://abclonal.com.cn/catalog/A3155;
Anti-PRMT6 antibody, Cat# 14641S, https://www.cellsignal.cn/products/primary-antibodies/prmt6-d5a2n-rabbit-mab/14641?site-search-type=Products&N=4294956287&Ntt=14641s&fromPage=plp&_requestid=4912009;
Anti-H3 antibody, Cat# ab1791, https://www.abcam.cn/histone-h3-antibody-nuclear-marker-and-chip-grade-ab1791.html;
Anti-α-Tubulin antibody, Cat# T6199, https://www.sigmaaldrich.cn/CN/zh/product/sigma/t6199;
Anti-GPX4 antibody, Cat# A11243, https://abclonal.com.cn/catalog/A11243;
Anti-GST antibody, Cat# AE006, https://abclonal.com.cn/catalog/AE006;
Anti-PARP antibody, Cat# 9542, https://www.cellsignal.cn/products/primary-antibodies/parp-antibody/9542?site-search-type=Products&N=4294956287&Ntt=9542&fromPage=plp&_requestid=4776062;
Anti-His antibody, Cat# AE003, https://abclonal.com.cn/catalog/AE003;
Anti-4 Hydroxynonenal antibody, Cat# ab46545, https://www.abcam.cn/4-hydroxynonenal-antibody-ab46545.html;
Anti-GAPDH antibody, Cat# sc-25778, https://www.scbt.com/p/gapdh-antibody-fl-335?requestFrom=search;
Anti-JNK antibody, Cat# 9252, https://www.cellsignal.cn/products/primary-antibodies/sapk-jnk-antibody/9252?site-search-type=Products&N=4294956287&Ntt=9252&fromPage=plp&_requestid=4776403;
Anti-p-JNK antibody, Cat# 9255, https://www.cellsignal.cn/products/primary-antibodies/phospho-sapk-jnk-thr183-tyr185-g9-mouse-mab/9255?site-search-type=Products&N=4294956287&Ntt=9255&fromPage=plp&_requestid=4909988;
Anti-p38 antibody, Cat# 9212, https://www.cellsignal.cn/products/primary-antibodies/p38-mapk-antibody/9212?site-search-type=Products&N=4294956287&Ntt=9212&fromPage=plp&_requestid=4910743;

Anti-p-p38 antibody, Cat# 9211, https://www.cellsignal.cn/products/primary-antibodies/phospho-p38-mapk-thr180-tyr182-antibody/9211?site-search-type=Products&N=4294956287&Ntt=9211&fromPage=plp&_requestid=4911422;
Goat-anti-Mouse HRP, Cat# ZB-2305, http://www.zsbio.com/product/ZB-2305;
Goat-anti-Rabbit HRP, Cat# ZB-2306, http://www.zsbio.com/product/ZB-2306;
Goat-anti-Mouse Alexa Fluor 594, Cat# ZF-0513, http://www.zsbio.com/product/ZF-0513;
Goat-anti-Mouse FITC, Cat# ZF-0312, http://www.zsbio.com/product/ZF-0312;
Goat anti-Mouse IgG (H+L) Secondary Antibody, DyLight 405, Cat# 35501BID, https://www.thermofisher.cn/cn/zh/antibody/product/Goat-anti-Mouse-IgG-H-L-Secondary-Antibody-Polyclonal/35501BID.
Purified rabbit anti-PtpA antibody has been validated by immunoblotting of whole bacterial lysates of wild-type or ptpA-depleted Mtb strains. A specific band at the predicted size of PtpA can be detected by rabbit anti-PtpA antibody .

Eukaryotic cell lines

Policy information about cell lines and Sex and Gender in Research

Cell line source(s)	HEK293T cells (ATCC CRL-3216, RRID: CVCL_0063).<br>A549 cells (ATCC CCL-185, RRID: CVCL_0023).<br>U937 cells (ATCC CRL-1593.2, RRID: CVCL_0007).
Authentication	No further authentication was made.
Mycoplasma contamination	All used cell lines were routinely tested and confirmed negative for mycoplasma contamination.
Commonly misidentified lines (See ICLAC register)	No commonly misidentified cell lines were used in this study.

Animals and other research organisms

Policy information about studies involving animals; ARRIVE guidelines recommended for reporting animal research, and Sex and Gender in Research

Laboratory animals	Male BALB/c mice (6–8 weeks) purchased from Vital River were used in this study. All the mice were housed under SPF condition (12 h light/dark cycle, 50% relative humidity, between 25 and 27°C) with free access to food and tap water.
Wild animals	No wild animals were used in the study.
Reporting on sex	We used male BALB/c mice in this study, since male mice are more susceptible to Mtb infection compared with female mice.
Field-collected samples	No field-collected samples were used in the study.
Ethics oversight	All experimental protocols were performed in accordance with the instructional guidelines of the China Council on Animal Care, and were approved by the Biomedical Research Ethics Committee of the Institute of Microbiology, Chinese Academy of Sciences (SQIMCAS2018005).

Note that full information on the approval of the study protocol must also be provided in the manuscript.

Flow Cytometry

Plots

Confirm that:

☒ The axis labels state the marker and fluorochrome used (e.g. CD4-FITC).

☒ The axis scales are clearly visible. Include numbers along axes only for bottom left plot of group (a 'group' is an analysis of identical markers).

☒ All plots are contour plots with outliers or pseudocolor plots.

☒ A numerical value for number of cells or percentage (with statistics) is provided.

Methodology

Sample preparation	Single cell suspensions were incubated with BODIPY (581/591) C11 probe in PBS, followed by flow cytometry analysis. The detailed experimental procedures were described in the Methods section.
Instrument	BD LSRFortessa SORP.
Software	FlowJo v10 was used for data analysis.
Cell population abundance	Flow-assisted cell sorting was not used. Populations were counted until 20,000 cells to analyze further population.
Gating strategy	Populations were gated for single cells based on forward (FSC) and side scatter (SSC). Cells labeled with BODIPY (581/591)

Gating strategy	C11 probe were analyzed in the FL1 channel. Cells unlabeled with BODIPY (581/591) C11 probe were used as negative control.

☒ Tick this box to confirm that a figure exemplifying the gating strategy is provided in the Supplementary Information.
