# Supplementary material for: A mycobacterial effector promotes ferroptosis-dependent pathogenicity and dissemination
Source: Nat Commun. 2023 Mar 17;14:1430. doi: 10.1038/s41467-023-37148-x (PMC10023711; doi:10.1038/s41467-023-37148-x)
Supplement: Supplementary file 8 — Source Data [file 41467_2023_37148_MOESM8_ESM.zip › Original Uncropped Scans Of Blots (2023.2.7).pdf]

Figure 1h

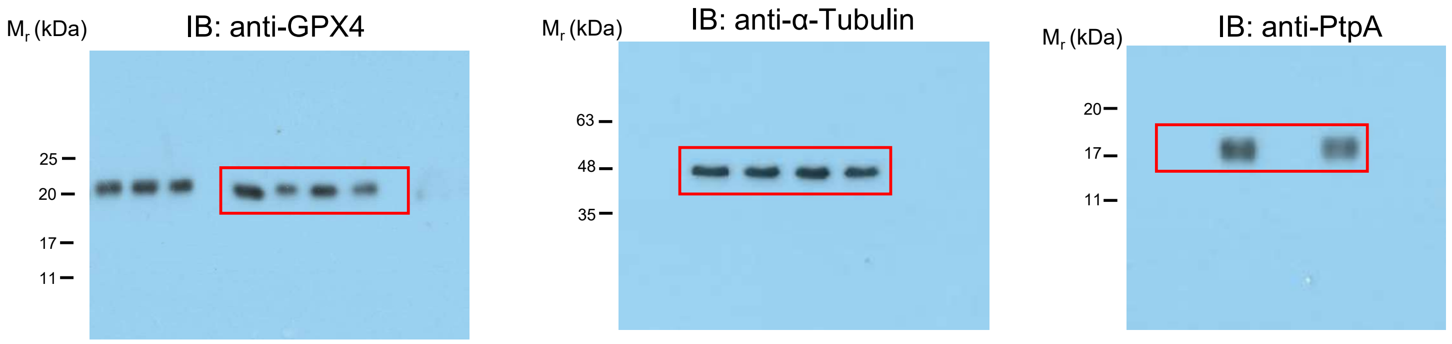

Figure 2a

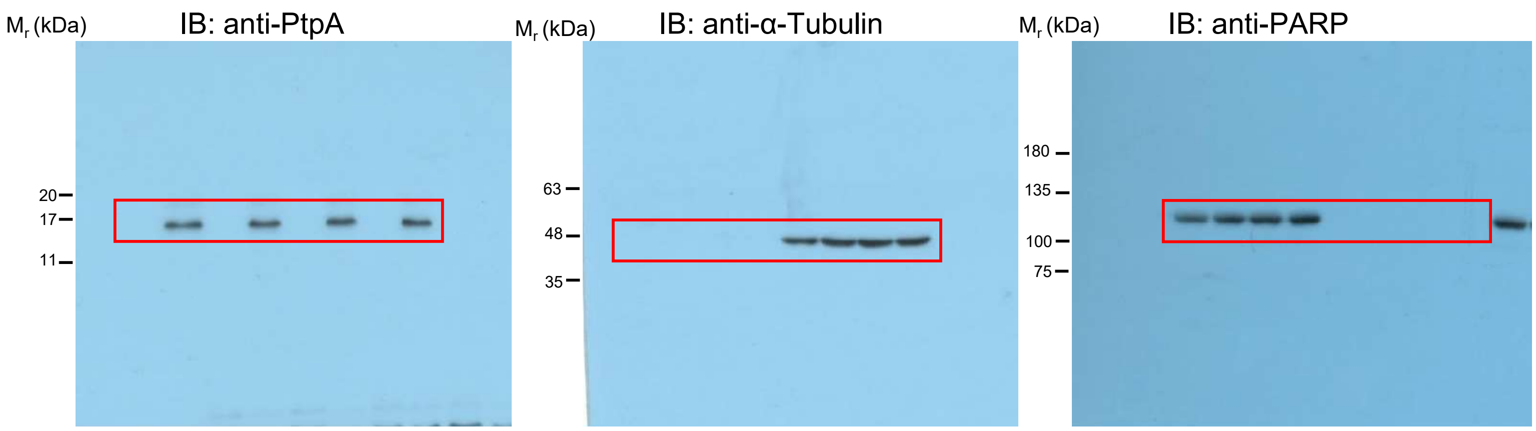

Figure 2c

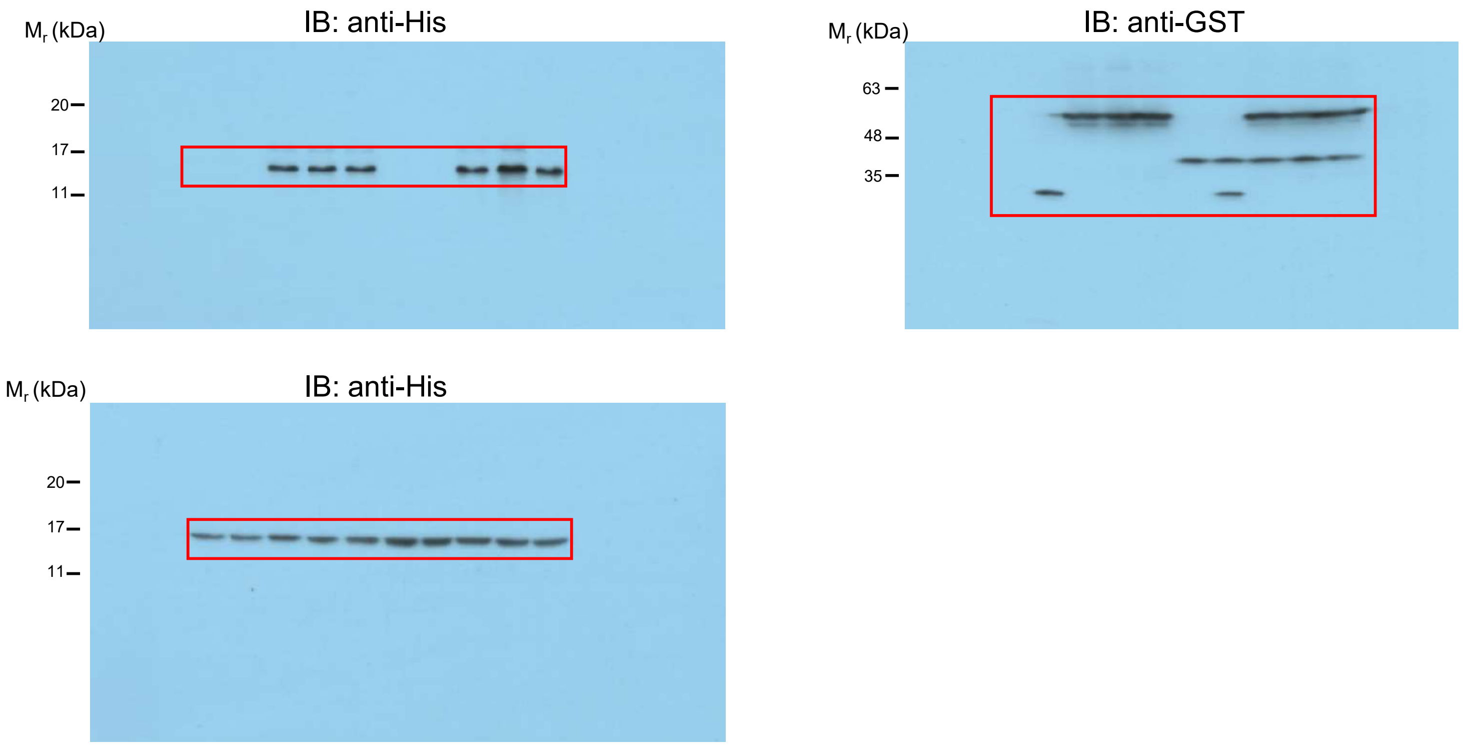

Figure 2f

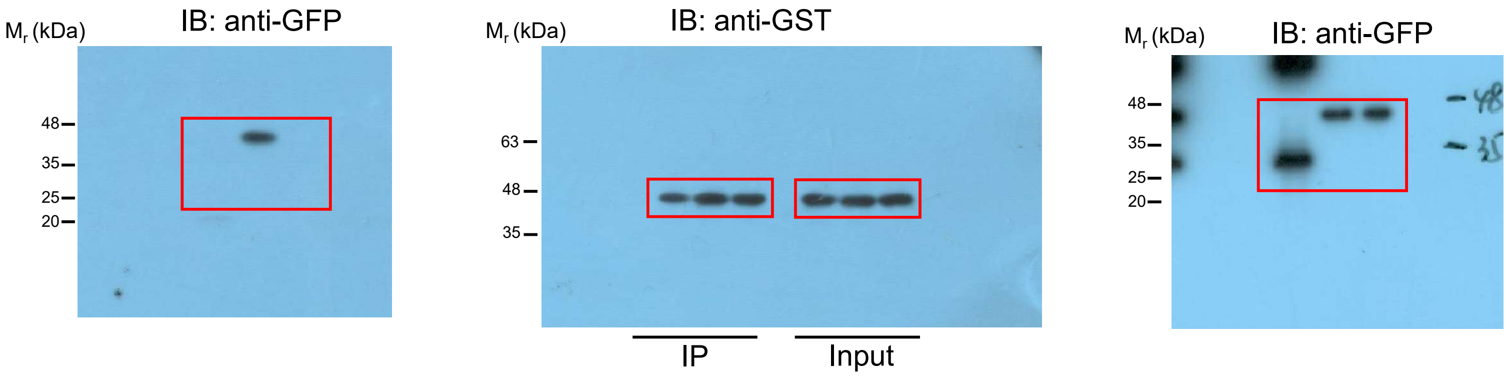

Figure 3a

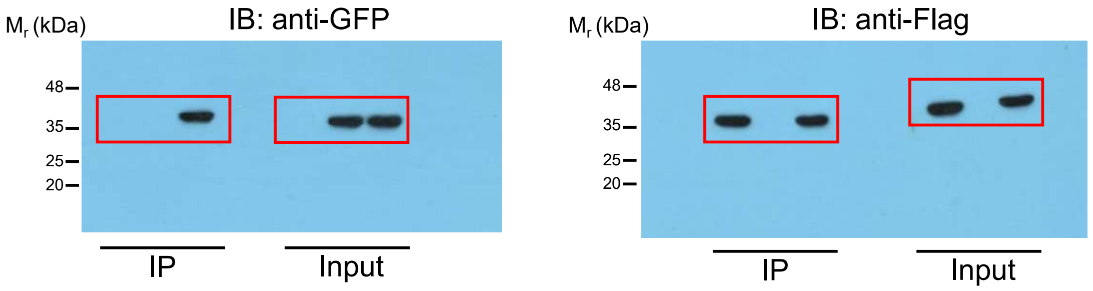

Figure 3d

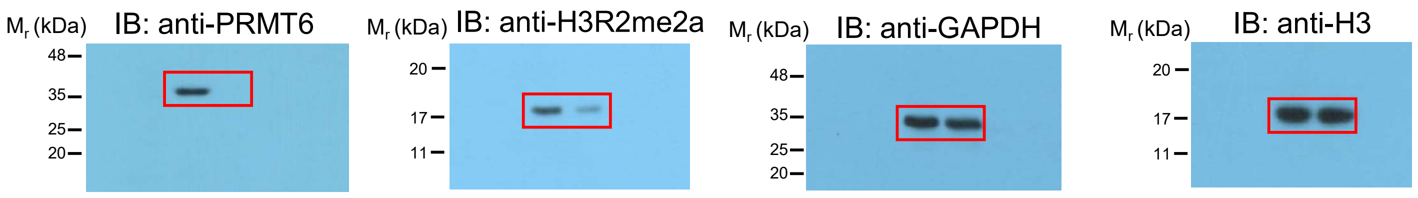

Figure 4a

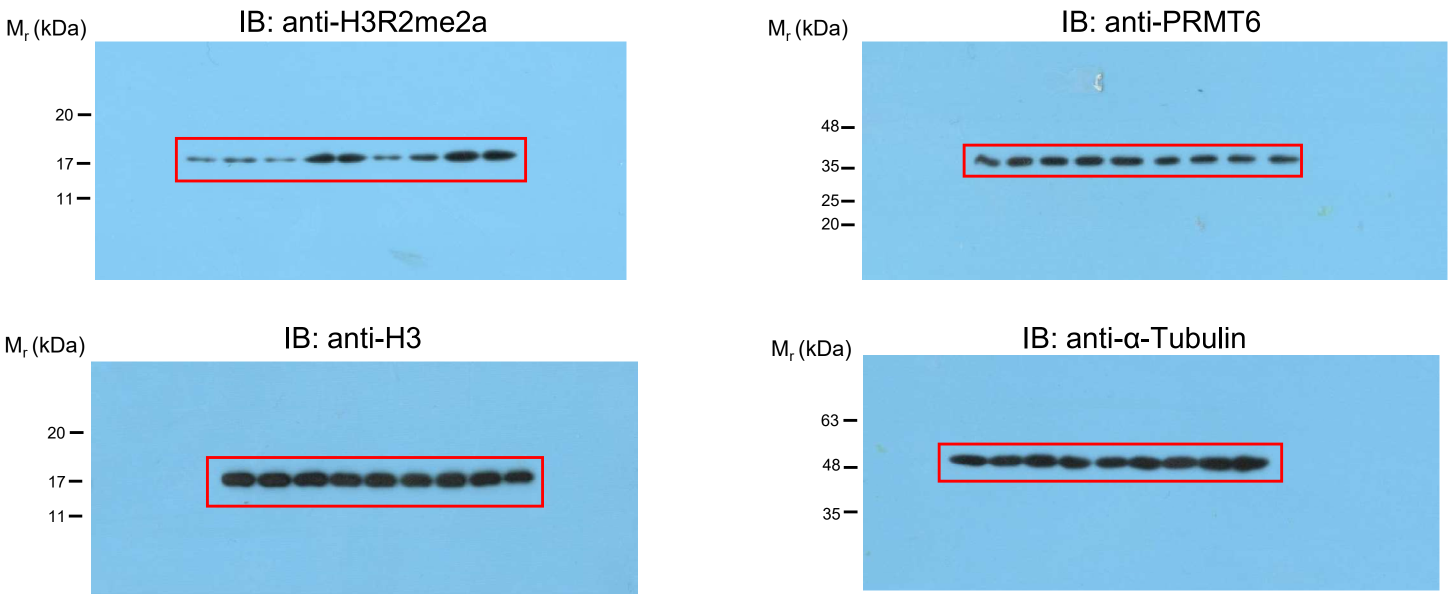

Figure 4b

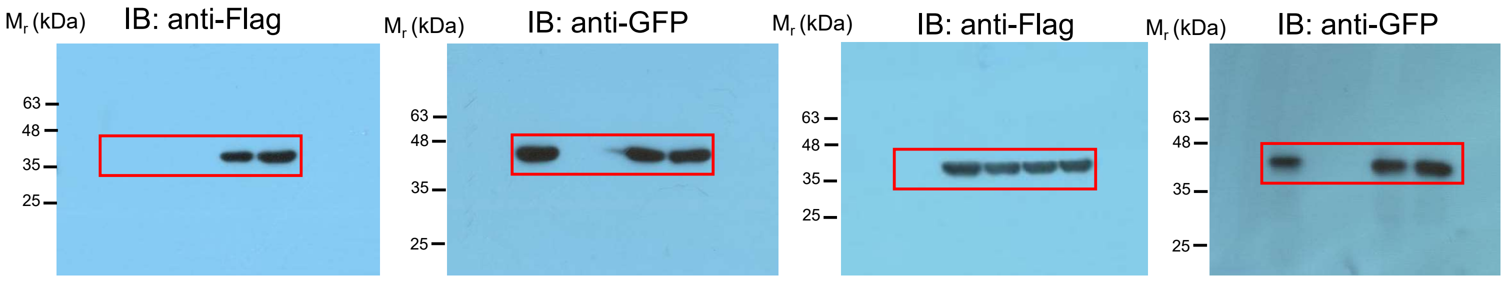

Figure 4j

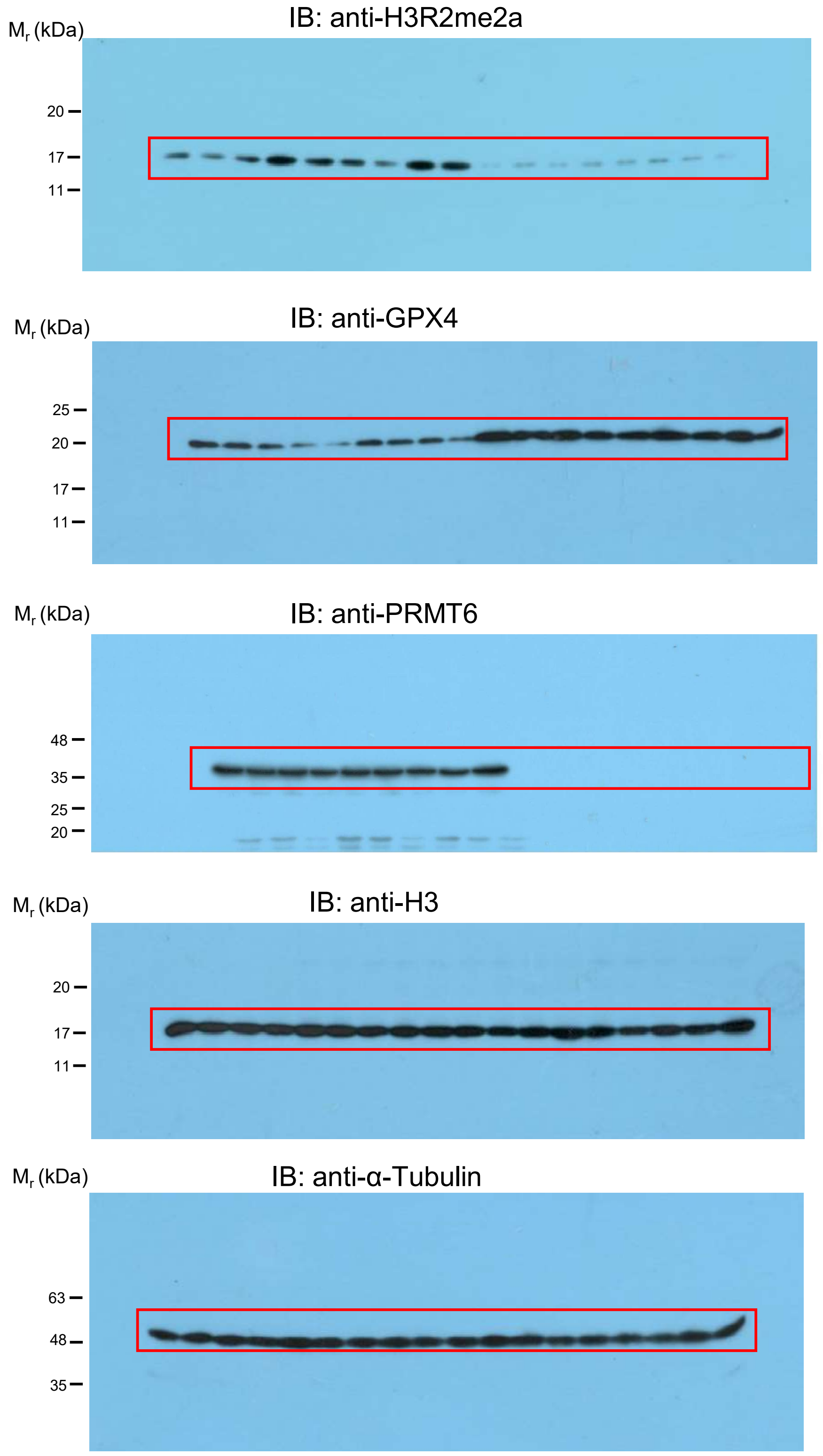

Figure 5a

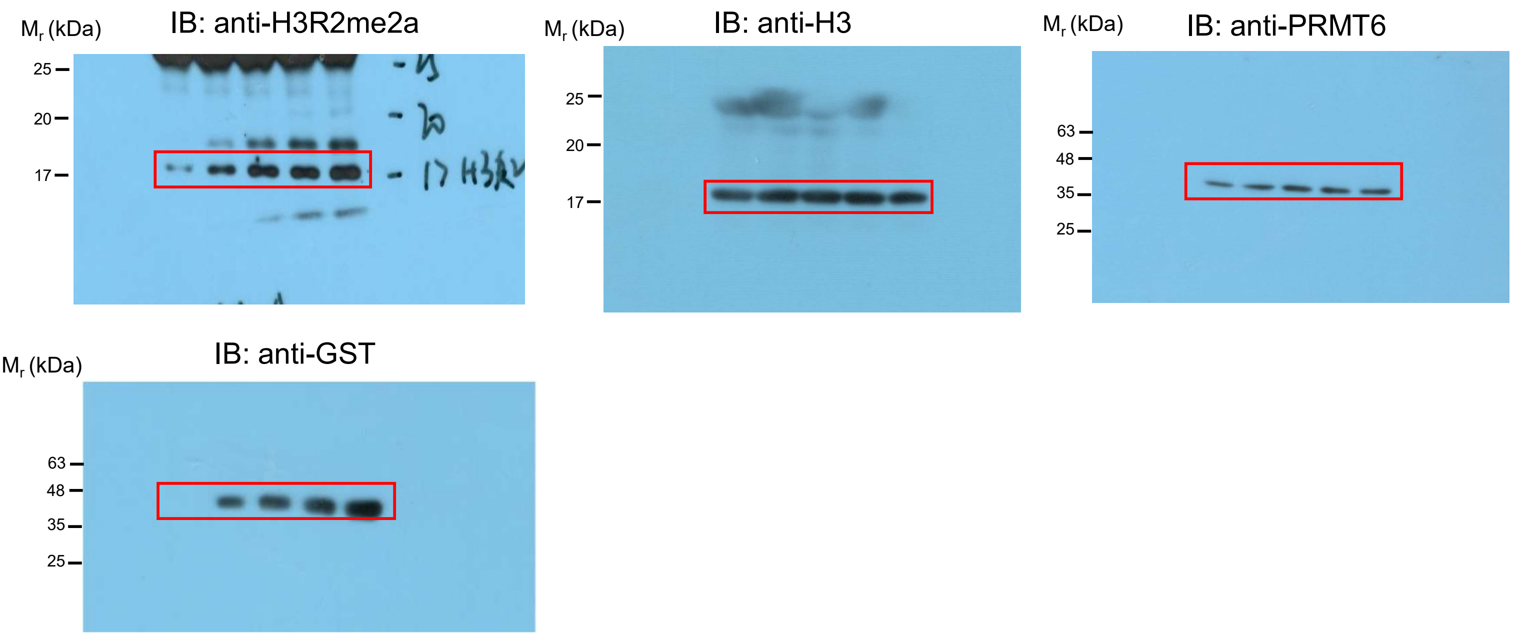

Figure 5c

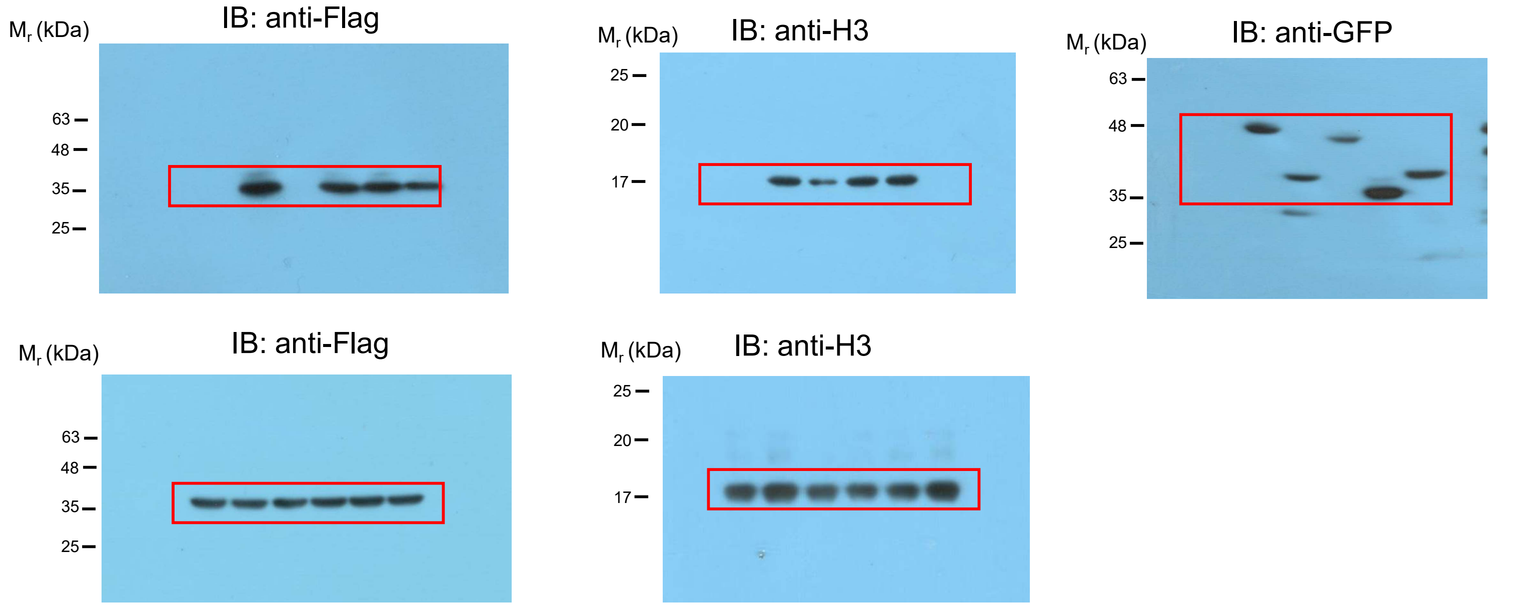

Figure 5d

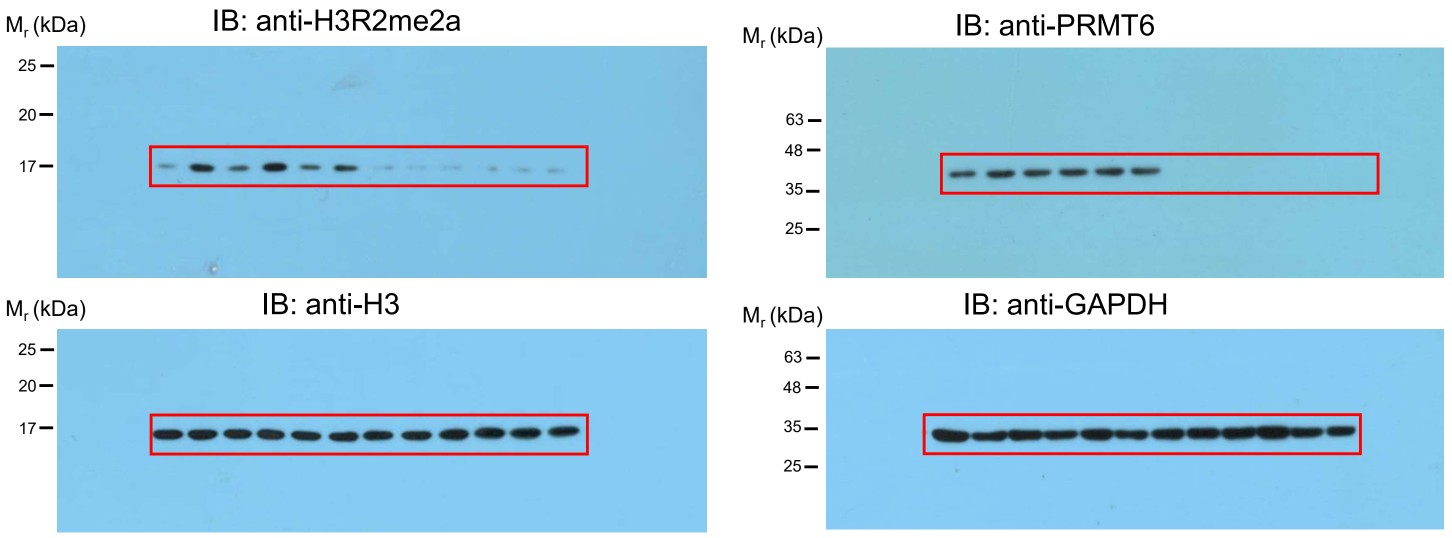

Figure S2a

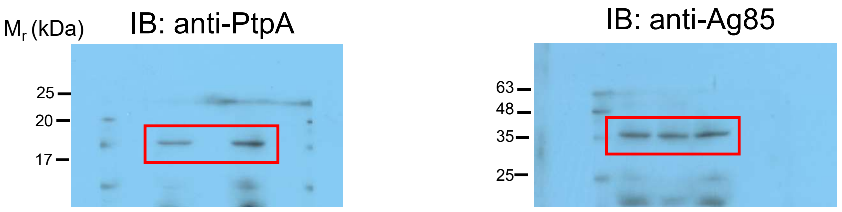

Figure S2i

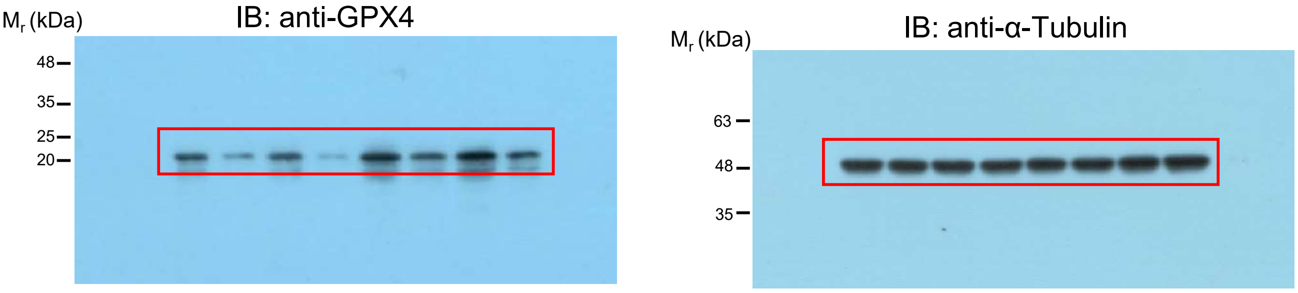

Figure S5b

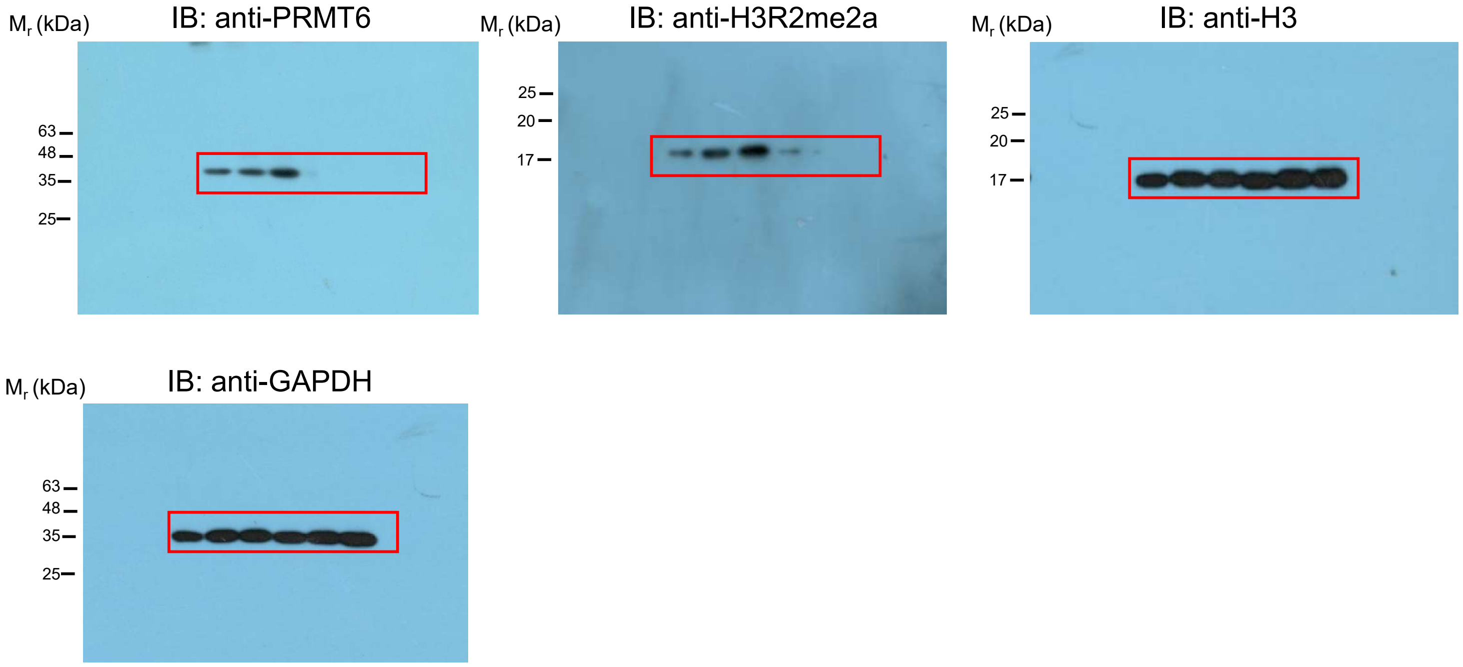

Figure S5c

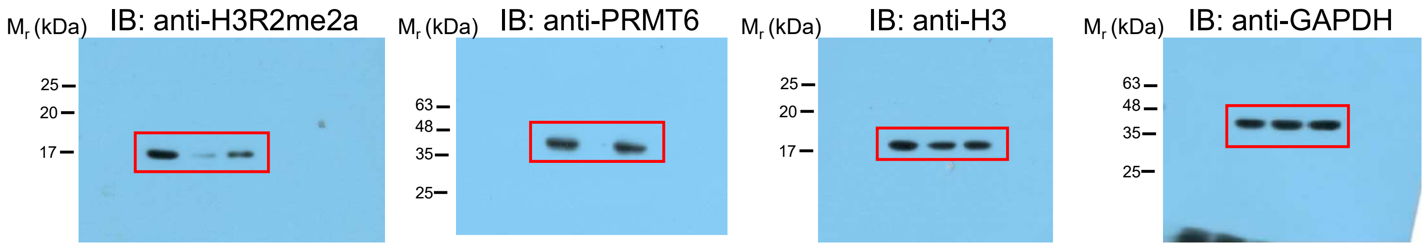

Figure S6b

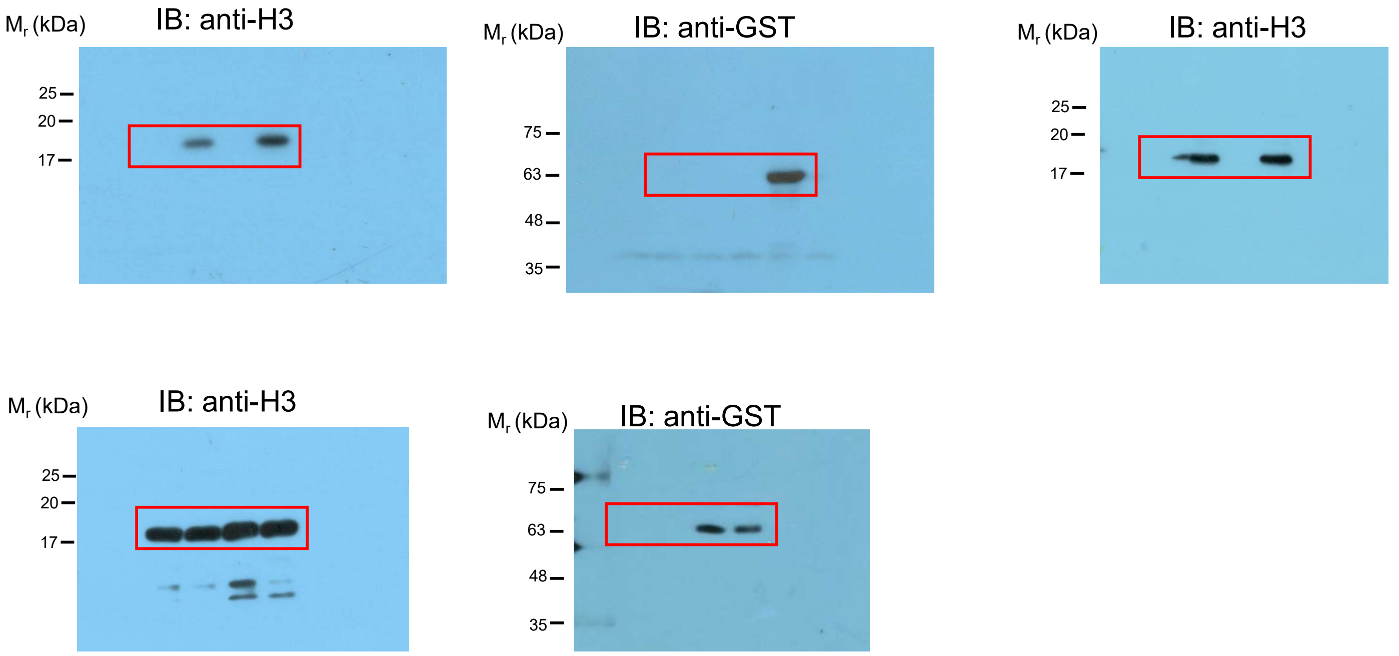

Figure S6e

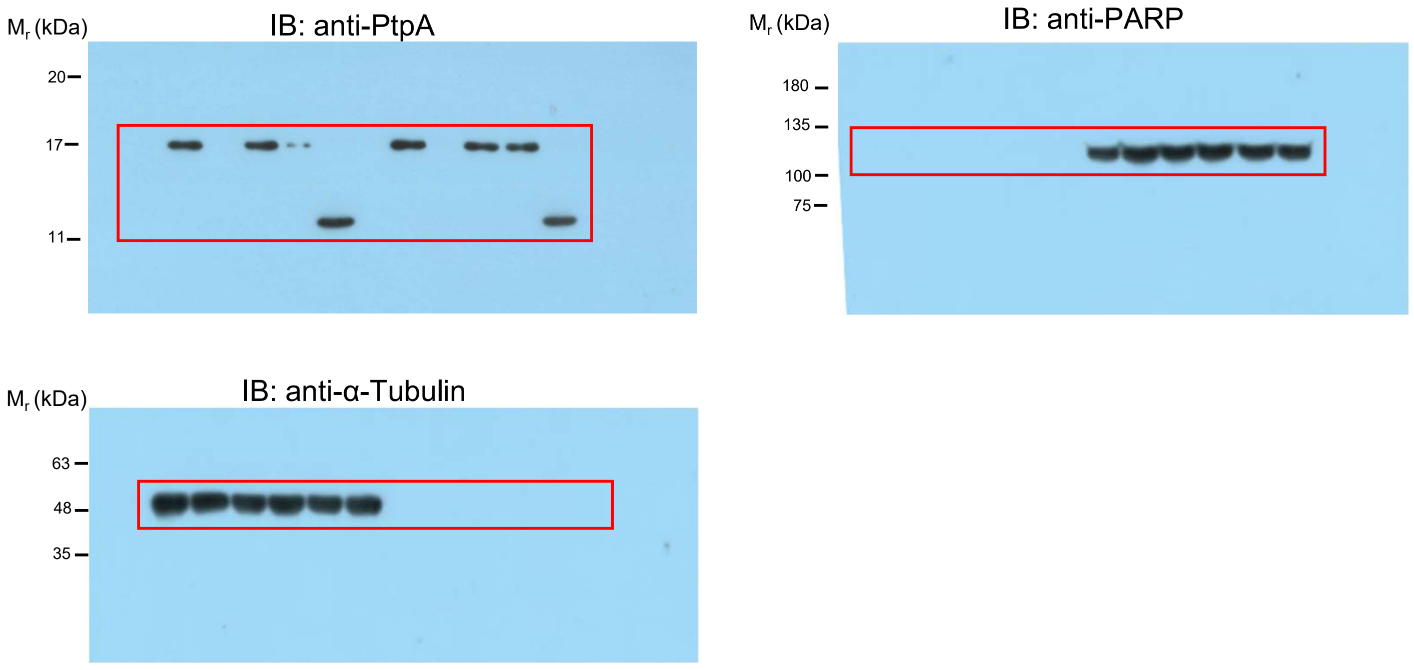

Figure S6h

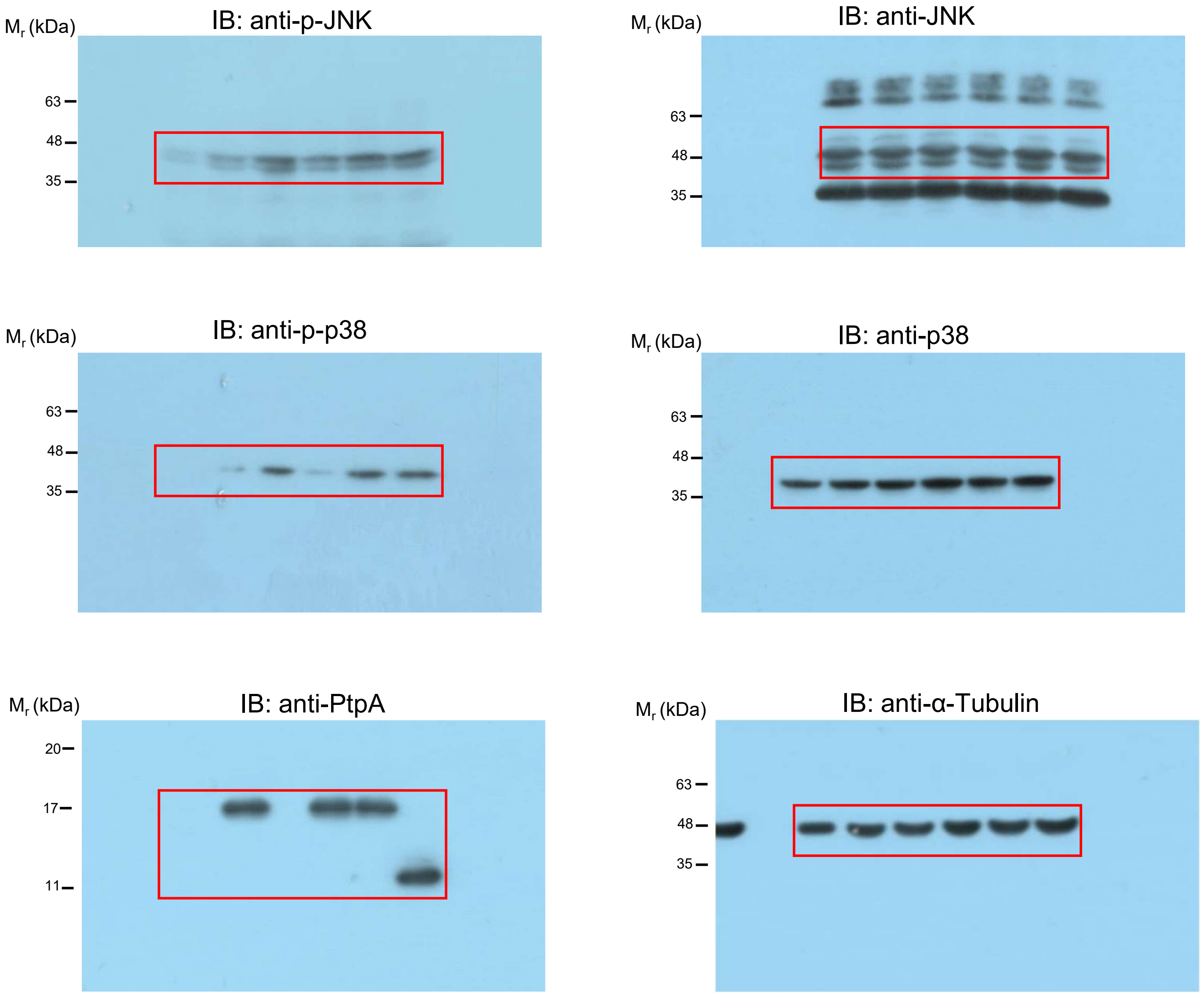
IB: anti-p-JNK
Mr (kDa)
63
48
35
IB: anti-JNK
Mr (kDa)
63
48
35
IB: anti-p-p38
Mr (kDa)
63
48
35
IB: anti-p38
Mr (kDa)
63
48
35
IB: anti-PtpA
Mr (kDa)
20
17
11
IB: anti-α-Tubulin
Mr (kDa)
63
48
35
